# Development of Stereo NIR-II Fluorescence Imaging System for 3D Tumor Vasculature in Small Animals

**DOI:** 10.3390/bios12020085

**Published:** 2022-01-30

**Authors:** Shih-Po Su, Syue-Liang Lin, Yang-Hsiang Chan, Yi-Jang Lee, Yun-Chen Lee, Pin-Xuan Zeng, Yi-Xuan Li, Muh-Hwa Yang, Huihua Kenny Chiang

**Affiliations:** 1 Department of Biomedical Engineering, National Yang Ming Chiao Tung University, Taipei 11221, Taiwan; maxsu0629.be06@nycu.edu.tw (S.-P.S.); SLR@nycu.edu.tw (S.-L.L.); yunyeny.be09@nycu.edu.tw (Y.-C.L.); estherzeng@gm.ym.edu.tw (P.-X.Z.); 2 Biomedical Engineering Research and Development Center, National Yang Ming Chiao Tung University, Taipei 11221, Taiwan; 3 Department of Applied Chemistry, National Yang Ming Chiao Tung University, Hsinchu 30010, Taiwan; yhchan@nycu.edu.tw (Y.-H.C.); lyx101838@gmail.com (Y.-X.L.); 4 Department of Biomedical Imaging and Radiological Sciences, National Yang Ming Chiao Tung University, Taipei 11221, Taiwan; yjlee2@nycu.edu.tw; 5 Institute of Clinical Medicine, National Yang Ming Chiao Tung University, Taipei 11221, Taiwan; mhyang2@nycu.edu.tw

**Keywords:** NIR-II fluorescence imaging, stereo imaging, polymer dots, reconstruction, tumor vasculature

## Abstract

Near-infrared-II (NIR-II, 1000–1700 nm) fluorescence imaging boasts high spatial resolution and deep tissue penetration due to low light scattering, reduced photon absorption, and low tissue autofluorescence. NIR-II biological imaging is applied mainly in the noninvasive visualization of blood vessels and tumors in deep tissue. In the study, a stereo NIR-II fluorescence imaging system was developed for acquiring three-dimension (3D) images on tumor vasculature in real-time, on top of the development of fluorescent semiconducting polymer dots (IR-TPE Pdots) with ultra-bright NIR-II fluorescence (1000–1400 nm) and high stability to perform long-term fluorescence imaging. The NIR-II imaging system only consists of one InGaAs camera and a moving stage to simulate left-eye view and right-eye view for the construction of 3D in-depth blood vessel images. The system was validated with blood vessel phantom of tumor-bearing mice and was applied successfully in obtaining 3D blood vessel images with 0.6 mm- and 5 mm-depth resolution and 0.15 mm spatial resolution. The NIR-II stereo vision provides precise 3D information on the tumor microenvironment and blood vessel path.

## 1. Introduction

Fluorescence imaging is an essential tool for drug distribution monitoring, cancer-targeting, or tumor therapy preclinical studies [1,2,3]. By labeling tumor tissues with a fluorescent dye, the fluorescence imaging system can record fluorophore distribution noninvasively [4]. In visible and NIR-I windows (400–900 nm), the imaging depth is restricted by high tissue scattering and absorption [5,6,7]. The imaging depth in the NIR-I region is 1–3 mm [8,9,10], but the imaging depth can exceed 5 mm in the NIR-II region due to lower tissue scattering and autofluorescence [11,12]. Light scattering suppression and low tissue absorption facilitate in vivo fluorescence imaging visualization of blood vessels with a high signal-to-noise ratio (SNR) [13,14], thereby detecting a tumor region according to blood vessel maturation or tumor-induced angiogenesis [15]. NIR-II imaging agents have been applied in optical angiography for visualizing blood vessel structure [16,17,18,19,20,21]. In our previous study, NIR-II fluorescent semiconducting polymer dots (IR-TPE Pdots) were developed to obtain high SNR NIR-II blood vessel imaging [22,23].

With angiogenesis being an omen for tumor formation, angiography has been applied in the diagnosis of various diseases associated with the malformation of blood vessels for over a century. In recent years, 3D imaging of vasculature of tumor mass has been applied in tumor diagnosis and therapy extensively. Preclinical/clinical optical imaging modalities have been developed for 3D imaging of vasculature, including optical conference and photoacoustic tomography (OCT/PAT) [24,25,26]. A recent report has confirmed the feasibility of using a cross-modality imaging pipeline to visualize and assess tumor vasculature in animals [27]. Tumor vasculature imaging is a key tool in evaluating tumor microenvironment and drug delivery, while 3D imaging modality is essential for a panoramic view of angiogenesis in tumor tissues in a preclinical study.

In recent years, the NIR-II fluorescence imaging system has evolved from 2D imaging to 3D imaging [28,29,30,31,32,33]. The 3D image of vascular structure is instrumental in understanding blood supply and detecting tumor tissue. Researchers have reconstructed 3D vessel structure with NIR-II confocal microscopy [28,29,30] and photoacoustic (PA) imaging methods [31,32,33]. Zhang et al. developed a bright fluorescent probe with ∼1600 nm emission and obtained a 3D image of mouse hind limb vessels via NIR-II fluorescence confocal imaging [29]. Wan et al. showed a 3D image of brain vasculatures obtained with a home-built NIR-II confocal setup [30], with a low penetration depth (less than 1.3 mm) [34]. In addition, it takes a much longer time to scan larger samples. Therefore, some researchers have resorted to NIR-II PA imaging to obtain large penetration depth images. Laufer et al. built 3D PA imaging of subcutaneous tumors with ~100 μm resolution with a photoacoustic computed tomography system [32]. Although NIR-II PA imaging boasts a 1–2 cm penetration depth and 0.1 mm spatial resolution, the NIR-II PA system has limited application, due to its long operating time [35]. Some researchers have proposed the use of binocular stereo vision to secure vessel depth information with high measurement accuracy [36,37]. Chen et al. produced a 3D image of the forearm subcutaneous vein via NIR stereo imaging with a compact venipuncture robot [36], an approach that boasts high cost-effectiveness and fast measurement [37].

The study developed a preclinical NIR-II 3D vessel stereo imaging system for in-vivo whole-body imaging and tumor vessel network for application in small animal research, for the first time ever. Self-made NIR-II dye IR-TPE Pdots were used for noninvasive fluorescence images of vessel structure and tumor vasculature, as IR-TPE Pdots are capable of over 3 mm deep penetration and the provision of the image on blood vessel distribution in mouse abdomen. The new system can materialize high-contrast tumor vasculature imaging, with up to 0.6 mm depth resolution.

## 2. Materials and Methods

### 2.1. Experimental Apparatus

**Figure 1:**
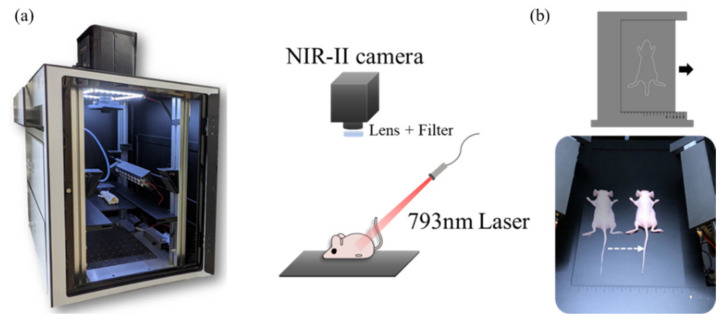
A home-built NIR-II fluorescence stereo imaging setup. (**a**) Photograph and schematic diagram of the epi-illumination NIR-II fluorescence stereo imaging system (**b**) Photograph of the one-dimensional moving stage for mice 3D vasculature reconstruction.

Figure 1a shows the NIR-II fluorescence stereo system, featuring epi-illumination geometry and fiber-based configurations with a fluorescence signal excited by a 793 nm continuous-wave fiber laser (CNI laser, FC-W-793). Then, light emitted from fiber (MHP550L02, Thorlabs, Newton, NJ, USA) was transmitted via a ground glass diffuser (Thorlabs, DG10-600-MD) providing uniform illumination in the irradiation area (50–70 mW/cm^2^). For whole-body imaging of the mouse, long-pass (LP) filters (FELH1100, FELH1300, and FELH1400, Thorlabs) were employed, while in vivo images were acquired using a cooled InGaAs camera (NIRvana 640, Princeton Instruments; 640 × 512 pixels, response 900–1700 nm) with a short-wave infrared C-mount zoom lens (LM35HC-SW, Kowa, Tokyo, Japan). The working temperature of the InGaAs camera was −80 °C, the gain was set in high mode, and the analog-to-digital conversion rate was set at 2 or 10 MHz. A camera and a one-dimension moving stage were used for NIR-II stereo imaging (Figure 1b). The images were acquired with LightField software (Princeton Instruments, Trenton, NJ, USA) and analyzed with MATLAB (MathWorks, 2020).

### 2.2. Intralipid^®^ Phantom

Where 1% Intralipid^®^ solution was prepared by diluting 20% Intralipid^®^ (Sigma-Aldrich, St. Louis, MO, USA) with deionized water. A 3D printing black box filled with 1% Intralipid^®^ solution was placed on a plate. Glass capillary tubes (OD = 1.5 mm/ID = 1.1 mm) filled with IR-TPE Pdots were immersed in the Intralipid^®^ solution. IR-TPE Pdots were synthesized according to the method in our previous study [22,23]. Capillaries were immersed at depths from 1 to 6 mm from the surface. Supplementary Tables S1 and S2 summarize the parameter sets of imaging (excitation source, optical filters, and integration times). All the images were collected with a homemade NIR-II imaging system. The average fluorescence intensity was captured from the same region of interest (ROI). Statistical analysis results were obtained using Origin 8.0.

### 2.3. Animal Experiments

Mouse tongue carcinoma 4NQO-induced cancer cell line (MTCQ1) was established from the oral squamous cell carcinoma (OSCC) cell line, obtained from the JCRB cell bank [38]. Cell lines, kept in RPMI (GIBCO^®^ Invitrogen Inc., Carlsbad, CA, USA) medium with 10% fetal bovine serum (HyClone^®^ Thermo, Waltham, MA, USA), 50 µg/mL of penicillin/streptomycin (Sigma-Aldrich Co., St. Louis, MO, USA), 2 mM of l-glutamine (Sigma-Aldrich Co., St. Louis, MO, USA), were incubated at a 37 °C in a humidified incubator with 5% CO^2^ and passaged every two days.

Purchased from the National Laboratory Animal Center (Taipei, Taiwan), BALB/c nude female mice (5–6 weeks, 18–22 g) were housed under conditions of controlled temperature (22 ± 1°C) and a 12-h light/dark cycle with free access to food and water. In the in vivo experiments, approved by the Institutional Animal Care and Use Committee of the National Yang Ming Chiao Tung University (approval number: 1100509), for MTCQ1 tumor establishment, a total of 1 × 10^6^ MTCQ1 cells were injected into the right hindlimb of BALB/c female nude mice subcutaneously. Cells were 1:1 mixed with Matrigel (BD Biosciences), resulting in a total volume of 200 μL.

### 2.4. In Vivo NIR-II Fluorescence Imaging

When the tumor of the MTCQ1 tumor-bearing nude grew to 6 mm in diameter, the mice were intravenously injected with IR-TPE Pdots (200 μL, 5 mg/mL) (n = 3). Then, the mice were anesthetized with 2% isoflurane to minimize the effect of respiratory movement. The in vivo NIR-II fluorescence imaging experiments were conducted with a custom-made NIR-II imaging system with different filters (1100 nm LP, 1300 nm LP, 1400 nm LP) and a C-mount lens (35 mm) under the 793 nm laser irradiation.

### 2.5. NIR-II Stereo Fluorescence Imaging

**Figure 2:**
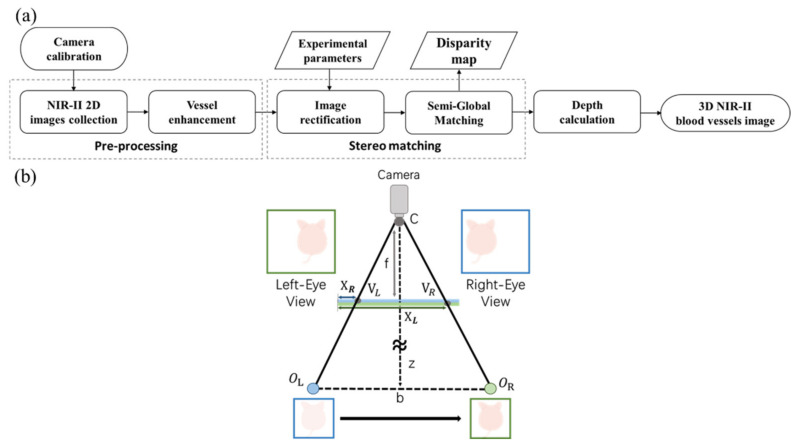
(**a**) The flow chart of the proposed NIR-II fluorescence stereo imaging system for 3D vascular imaging (**b**) Configuration scheme of our fluorescence stereo imaging system.

The principle in binocular stereo vision is to have two cameras focus on the same object. Binocular stereo vision boasts fast imaging speed and low cost. A stereo algorithm matches related patterns in the left-view image with the visible marks of the right-view image. Depth information can be obtained according to the disparity between corresponding points in the left and right view images [38,39]. Figure 2a shows the block diagram of our NIR-II camera stereo imaging, while Figure 2b exhibits the distance measurement of the stereo imaging system. Only a camera and a one-dimension moving stage were employed in building 3D vessels images from left-eye view and right-eye view, at a distance of 40 mm. Thus, the 3D tumor vasculature images can be built with NIR-II binocular stereo vision. Figure 2b shows the optical distance of the two images is b. Z is object distance, much larger than the focal length of camera f. V_L_ and V_R_ are the symmetrical positions of an object’s left- and right-view images. O_L_ and O_R_ are the object locations on the left and right, respectively. The following relationship results from a comparison of similar triangles CV_L_V_R_ and CO_L_O_R_:

(1) $$\frac{X_{L}-X_{R}}{f}\;\approx \;\frac{b}{Z}$$

In Equation (1), *b* is the distance between the optical centers of the two images, and *X_L_* and *X_R_* represent the abscissa values of the two images, respectively. *Z* represents the distance from camera C to the object, while *f* is the focal length of the camera. With *X_L_* − *X_R_* as disparity and *d* = *X_L_* − *X_R_*, then Equation (1) can be written as:

(2) $$Z\;\approx \frac{b*f}{d}$$

In Equation (2), after the distance between the cameras is measured, it only needs to determine the disparity for calculating the distance from the object to the camera [40]. The theoretical depth resolution (*D_r_*) of a stereo system at distance *Z* is calculated as follows:

(3) $$D_{r}\approx \;\frac{Z^{2}}{f*b}*\;△p$$

where *f* is the focal length of the camera, *b* is baseline length and △p is disparity error, which hinges on the accuracy of the algorithm matching the corresponding pixels in the two eye view images [41].

## 3. Results

### 3.1. Intralipid^®^ Phantom Validation

**Figure 3:**
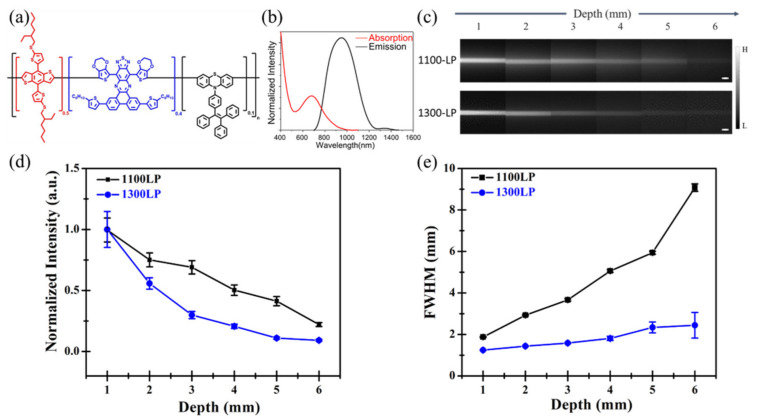
Intralipid ^®^ phantom study of IR-TPE Pdots in NIR-II window. (**a**) Chemical structures of IR-TPE Pdots. (**b**) Absorption (red line) and fluorescence (black line) spectra of IR-TPE Pdots (**c**) Fluorescence images of glass capillaries filled with IR-TPE Pdots (scale bar, 2 mm), (**d**) quantification results, and (**e**) wavelength-dependent FWHM were calculated for capillary glass tubes filled with IR-TPE Pdots at varying depths. Data derive from mean ± SD. n = 3 independent measurements.

Intralipid phantom study was meant to evaluate the imaging quality of IR-TPE Pdots in imaging ranging from 1100-LP (1100–1400 nm) to 1300-LP (1300–1400 nm) at different depths. The phantom was used for mimicking the optical characteristics of biological tissues in the NIR-II window. Figure 3a,b exhibit the chemical structure and absorption/emission spectra of IR-TPE Pdots, with a maximum emission at 1000 nm and up to 1400 nm in emission tail. Capillaries containing IR-TPE Pdots were irradiated from a 793 nm laser and emission was captured by the camera at different depths in the 1100-LP-1300-LP range.

Supplementary Tables S1 and S2 lists the detailed imaging parameters of each image—the laser energy was 25 mW/cm^2^ in the 1100-LP range and 50 mW/cm^2^ in the 1300-LP range. Figure 3c shows the NIR-II images of capillary tubes filled with IR-TPE Pdots aqueous solution, immersed in 1% Intralipid^TM^ at depths from 1 to 6 mm. The effect of light scattering became stronger, in line with depth increase in the 1100-LP range, while sharp margins of capillary tubes could be retained in the 1300-LP range, even at 5 mm depth. Figure 3d,e display quantitative fluorescence results.

The fluorescence intensities of capillary tubes in the 1300-LP range decreased much faster than that in the 1100-LP range (Figure 3d). The normalized intensities were halved at 4 mm depth in the 1100-LP range and at 2 mm depth in the 1300-LP range. Moreover, the spatial resolution of an optical imaging system was measured via full-width half maximum (FWHM) analysis, whose results for NIR-II capillary images at different depths are shown in Figure 3e. The FWHM values of capillary tubes in the 1100-LP range at depths 1~6 mm from the intralipid^®^ surface were 1.87 ± 0.02~9.08 ± 0.18 mm, higher than that in the 1300-LP range at different depths of 1 (1.25 ± 0.01 mm)~6 (2.44 ± 0.62 mm) mm.

### 3.2. NIR-II 2D In Vivo Whole-Body Fluorescence Imaging

**Figure 4:**
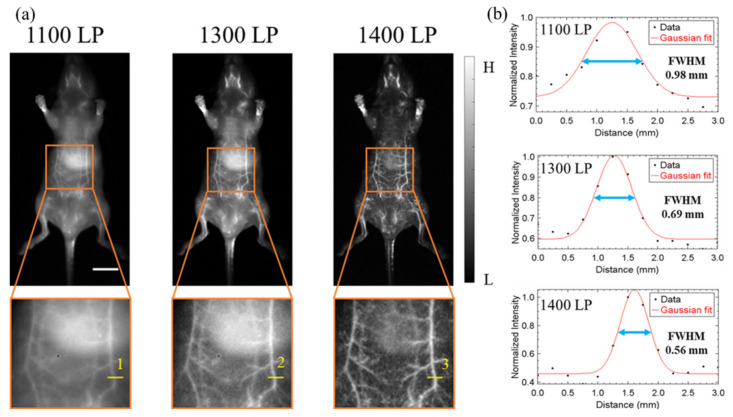
In vivo NIR-II whole-body fluorescence imaging of nude mice in supine positions (**a**) NIR-II bioimaging of mice after intravenous injection of IR-TPE Pdots (n = 3) with different LP filters (scale bar, 10 mm). (**b**) Fluorescence intensity signals (black points) and Gaussian fitting curve (solid red line) along yellow lines 1–3 in the NIR-II fluorescence image are shown in (**a**).

The imaging performance of IR-TPE Pdots was tested with noninvasive in vivo vascular imaging of mice (n = 3). Placed on the imaging platform in supine positions, mice were intravenously injected with 200 μL of 5 mg/mL aqueous solution of IR-TPE Pdots. NIR-II fluorescence signals with various filters (1100 nm LP, 1300 nm LP, 1400 nm LP) were obtained to study the light scattering effect in different NIR-II regions. The mice’s blood vessels are clearly visualized in all the NIR-II imaging with different spatial resolutions (Figure 4a). The fluorescence intensities of the tissues decreased, in line with longer wavelength collection. Figure 4b shows the FWHM Gaussian measurement of the vessels (marked by the yellow line). The FWHM values of belly vessels were 0.98 mm (1100 nm LP filter), 0.69 mm (1300 nm LP filter), and 0.56 mm (1400 nm LP filter), respectively. These results show that IR-TPE Pdots could be applied as fluorescence imaging agents in NIR-II imaging, using a 1400 nm LP filter to achieve high resolution.

### 3.3. NIR-II 3D Stereo Fluorescence Imaging of Vasculature Phantom

**Figure 5:**
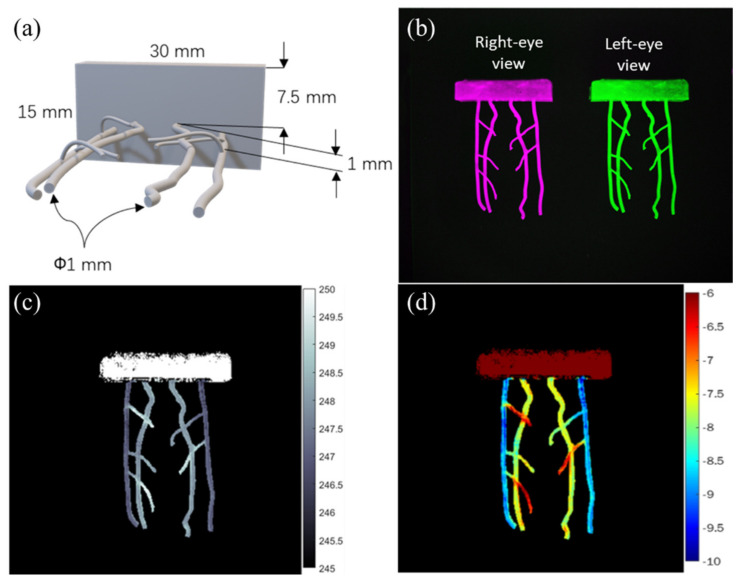
Results of blood vessel phantom study: (**a**) design of 3D printing phantom, (**b**) left eye- and right eye-view images, (**c**) disparity map of blood vessels phantom, and (**d**) depth map of blood vessels phantom.

Before in vivo study, the stereo imaging system was validated with a 3D printing blood vessel mimicking phantom, with 40 × 30 × 15 mm outer dimensions and (Figure 5a) and 1 mm diameter for each vessel. A customized stereo NIR-II imaging system was used to acquire left-eye view (green) and right-eye view (pink) images with a 40 mm baseline length for 3D stereo imaging (Figure 5b). The object distance of the system was 450 mm. Disparity map data were set with the semi-global matching (SGM) algorithm in the MATLAB programming environment [39,40]. The data were employed in calculating the depth map (Figure 5c). Figure 5d on the 3D blood vessels mimicking phantom shows that the vasculature phantom (imaging depth ~5 mm) was reconstructed correctly, with reconstruction depths ranging from −6 to −10 mm, displayed on a red-yellow-green-blue scale. The two blood vessels at depth 7.5 mm are in yellow color and the other two blood vessels at a depth of 8.5 mm are in blue color. In other words, the stereo imaging system can distinguish the upper vessels and lower vessels of the phantom.

### 3.4. NIR-II 3D In Vivo Stereo Fluorescence Imaging of Whole-Body Vasculature

**Figure 6:**
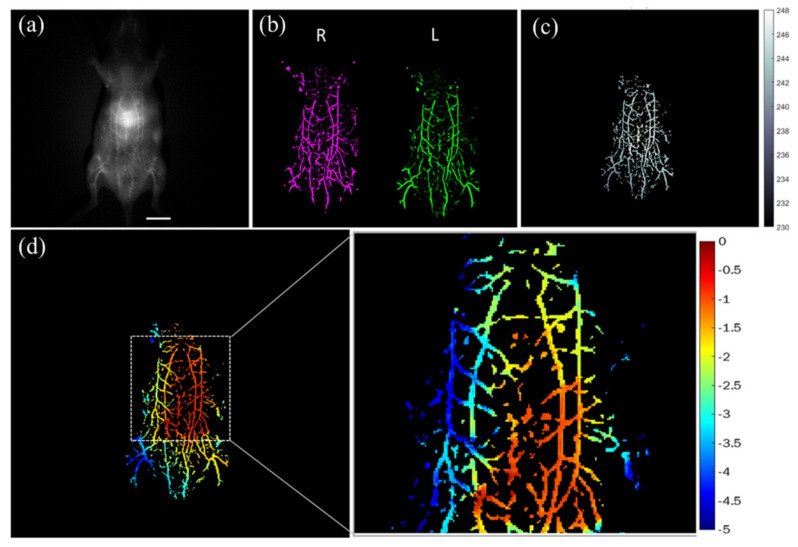
Results of in vivo 3D blood vessel study: (**a**) 2D NIR-II image (scale bar, 10 mm), (**b**) left and right vessel enhanced images, (**c**) disparity map of the mice’s blood vessels, and (**d**) depth map of the mice’s blood vessels in the abdominal region.

IR-TPE Pdots, which are suitable for long-term real-time imaging, were applied as in vivo imaging agents in acquiring high SNR abdomen 3D vessel images of mice (n = 3). In that case, 2D fluorescence imaging was acquired at 10 min post-injection with a 1300 nm LP filter (Figure 6a). A blood vessel enhancement algorithm based on the Hessian matrix filter was applied for the segmentation of blood vessels [41] by identifying the boundaries of blood vessels from the background and other non-vessel tissues. A custom-made stereo NIR-II imaging system containing one InGaAs camera and a moving stage was developed to acquire two eye view images with a 40 mm baseline (Figure 6b). A disparity map was calculated from the left-eye view and right-eye view and then converted to a depth map for achieving the 3D abdominal blood vessel imaging, as shown in Figure 6c,d. The result showed that the abdominal vascular network (imaging depth 0–5 mm) was correctly reconstructed. The red-yellow-green-blue color sequence was encoded to represent different vessel depths. Moreover, the main four abdominal vessels were displayed in different colors for different depths in the abdomen area of the mice with IR-TPE Pdots injection.

### 3.5. 3D NIR-II Fluorescent Angiography of Tumor-Bearing Mice

**Figure 7:**
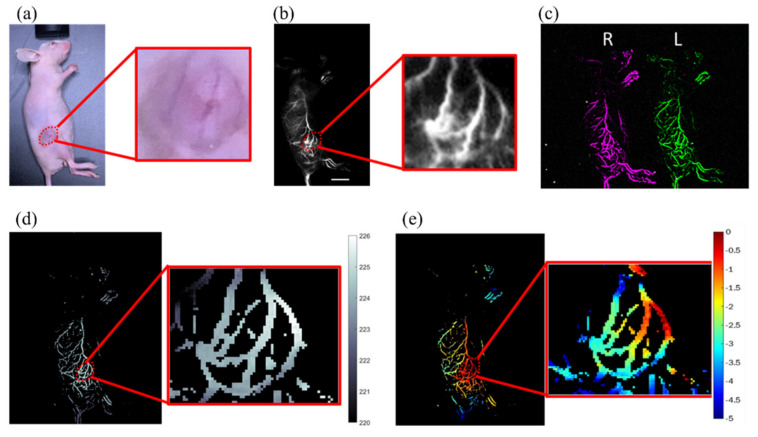
Results of in vivo blood vessel of tumor-bearing mice after intravenous injection of IR-TPE Pdots (inset: enlarged tumor region): (**a**) ambient image and (**b**) NIR-II fluorescence image (scale bar, 10 mm), (**c**) enhanced left- and right-vessel images, (**d**) disparity map of nude mice, and (**e**) depth map of nude mice with 6 mm tumor.

The study examined the 3D tumor blood vessel network of mice with a 6 mm tumor, which was designated as ROI. In three mice, a subcutaneous tumor was implanted in the leg/hindlimb. Figure 7 shows the 3D tumor vasculature reconstruction of the tumor-bearing mice injected with fluorescence agent IR-TPE Pdots. Ambient light and NIR-II fluorescence images are shown in Figure 7a,b. Then, the vascular enhancement and segmentation algorithm was employed to enhance the vascular morphology of the tumor area (ROI). Figure 7c shows the left- and right-eye view images. A disparity map deriving from the stereo matching process provides depth information for 3D reconstruction (Figure 7d). Figure 7e shows the depth map of tumor vessels. The reconstruction depths were represented in the depth range (0 to −5 mm) on a red-yellow-green-blue scale. According to the ambient image in Figure 7a, 3D blood vessels in the tumor area were restructured successfully, which showed depth information correctly.

## 4. Discussion

In the study, a 3D NIR-II fluorescence stereo imaging system was developed (Figure 1) and applied to small animals for NIR-II imaging, which was then used in the construction of the 3D models of abdominal blood vessel network and tumor vasculature. In addition to the advantages of fast imaging speed and low cost for binocular stereo vision, the system boasts easy-to-use and high accuracy, enabling the construction of the 3D structure of mice’s abdomen vessels.

The system has demonstrated the following imaging advantages: (1) NIR-II imaging has higher spatial resolution and SNR than the visible and NIR-I imaging; (2) the superior custom-developed IR-TPE Pdots enabled fluorescence imaging for noninvasive detection of blood flow and tumor metabolism; (3) the binocular stereo in-depth imaging system reconstructed whole-body vessel structure with a much wider viewing field than confocal microscopy; (4) fast imaging speed, due to the calculation of the disparity between the two different view images (Figure 2); (5) cost-saving, as it used only one NIR-II camera and a moving stage.

Before in vivo study, the penetration depths of the fluorescence of IR-TPE Pdots were determined by the Intralipid phantom. Fluorescence imaging of capillary tubes filled with IR-TPE Pdots was conducted, for comparing the difference between the 1100-LP and the 1300-LP ranges. Based on the phantom data, blurred edges were observed in the glass capillary tubes in the 1100-LP range, in contrast to sharp edges in the 1300-LP range (Figure 3c). Fluorescence intensity in the 1300-LP range decreased at a faster rate, due to the higher absorption coefficient of water beyond 1300 nm (Figure 3d). Moreover, the width of capillary images increased significantly, along with increased penetration depth in the 1100-LP range. There were apparent differences in the FWHM of these capillary tubes in the 1300-LP range, due to a much lower scattering coefficient. As the depth increased in the 1300-LP range, the FWHM of capillary tubes remained steady. The Intralipid phantom study showed that reliable fluorescent signals can be obtained at 5 mm penetration depth in biological tissues. In sum, the NIR-II system can yield deep small-animal imaging with high spatial resolution.

Figure 4 shows in vivo fluorescence images with different LP filters (1100 nm LP, 1300 nm LP, 1400 nm LP), along with imaging quality comparison. These data underscore the advantages of NIR-II imaging, including longer wavelength, high spatial resolution, and low background noise, indicating that IR-TPE Pdots and 1400 nm LP filter offer satisfactory fluorescence intensity and high spatial resolution for NIR-II imaging. Owing to reduced autofluorescence and the photon scattering of tissues at longer wavelengths, NIR-II imaging with a 1400 nm LP filter can achieve high-quality imaging for the blood vessel network in the belly region (Figure 4a). In addition, FWHM is small in longer wavelength emission channels, resulting in the advantage of lower photon scattering in the NIR-II window. NIR-IIa (1300–1400 nm) imaging boasts low background interference and higher imaging resolution, suitable for imaging on abdominal vessel structure.

Depth resolution, positively proportionate to the square of distance but inversely proportionate to baseline length (Equation (3)), is key to achieving high-quality stereo reconstruction [42]. Depth resolution can be improved by increasing baseline length or using lenses with larger focal lengths. At the object distance (450 mm) and baseline length (40 mm) of our system, depth resolution stood at 0.6 mm. The 3D depth maps of blood vessels mimicking phantom, healthy mice, and tumor-bearing mice are shown in Figure 5d, Figure 6d and Figure 7e. The shape of the phantom in the study was based on the abdominal vessels of mice for simulating real animal experiments and minimizing the number of animals in use (Figure 5a). The difference between the upper layer and lower layer of the phantom was 1 mm, sufficient for imaging. The two layers of the abdominal vessel phantom were correctly reconstructed in the 3D depth map (Figure 5d). For in vivo research, the noninvasive NIR-II stereo imaging system offers a convenient approach to the imaging of 3D vasculatures in the belly region of mice. The contrast of blood vessels was improved with a vascular enhancement and segmentation algorithm (Figure 6 and Figure 7). A high-contrast 3D blood vessel depth was made with enhanced blood vessel images. Results from blood-vessel phantom and healthy mice both show the two vessels in the upper layer and the other two vessels in the lower layer. In the tumor vascular depth map, the blood vessels’ in-depth range (−0.5 mm to −4 mm) was clustered along the edge of the tumor. Furthermore, the solid tumor is a spheroid that can induce angiogenesis to support the oxygen and nutrients for tumor growth. However, the vessels surrounding tumor mass are versatile, i.e., the tumor blood vessels can encompass the tumor in a 3D architecture. As angiogenesis will be initiated when the tumor size is larger than 1–2 mm [43], our system would provide sufficient depth resolution for early diagnosis of vessel formation in tumorigenesis. Additionally, angiogenesis is well-known as a hallmark for targeting in cancer therapy [44], and our system will be important for the assessment of different antiangiogenetic compounds by combining IR-TPE Pdots nanomaterials. In general, the 3D NIR-II stereo vision technique would be a powerful tool for estimating 3D abdominal vessel structure and tumor vessels with a large imaging area (48 cm^2^) and good spatial resolution (0.15 mm) at up to 5 mm penetration depth.

There is still some room for further improvement of the system. First, it is very challenging to distinguish tumor microvasculature with sub-mm depth resolution. The current depth resolution, 0.6 mm of the imaging system, could be further improved to 0.3 mm in-depth resolution by increasing baseline length or reducing object distance. Second, for optimizing the system’s imaging performance, we are developing a rotating stage to acquire more viewpoints than the moving stage. The multi-view stereo vision techniques may be useful for enabling accurate and 360-degree depth map estimation. Third, we are developing optimized algorithms for vascular extraction and quantification, including blood vessel depth estimation and neovascularization density measurement. Fourth, new fluorescent Pdots are under development to extend absorption and emission to the NIR-IIb (1500–1700 nm) region to boast better resolution and deeper tissue penetration than the current NIR-IIa (1300–1400 nm) region. Fifth, we developed a 3D NIR-II stereo imaging system using the subcutaneous tumor model, however, the orthotopic implantation of MTCQ cells is closer to the tumor microenvironment and natural neovascularization. Therefore, we would investigate NIR-II fluorescent angiography of MTCQ tumors by orthotopic implantation for tumor growth monitoring and therapy in a future study.

## 5. Conclusions

In the study, a stereo NIR-II fluorescence imaging system combining NIR-II fluorescence imaging and binocular stereo vision was developed and used in acquiring 3D tumor vascular and blood vessel images with 0.15 mm spatial resolution, 0.6 mm depth resolution, and 5 mm imaging depth. In sum, the NIR-II fluorescence stereo imaging technology promises a significant potential for imaging vascular tumor markers for early cancer detection.

## Data Availability

All data relevant to the study are included in the article.

## Supplementary Materials

The following are available online at https://www.mdpi.com/article/10.3390/bios12020085/s1, Table S1: the detailed parameter sets of fluorescence imaging in this study, Table S2: the integration time (ms) used for each fluorescence image of the phantom study.
