# Supplementary material for: Development of Stereo NIR-II Fluorescence Imaging System for 3D Tumor Vasculature in Small Animals
Source: Biosensors (Basel). 2022 Jan 30;12(2):85. doi: 10.3390/bios12020085 (PMC8869613; doi:10.3390/bios12020085)
Supplement: Supplementary file 1 [file biosensors-12-00085-s001.zip › biosensors-1546183-supplementary.pdf]

Supplementary Tables

Supplementary Table S1. the detailed parameter sets of fluorescence imaging in this study

Figures	Concentration	Laser ($mWcm^{-2}$)	Optical Filter	Integration time (ms)
Figure 3c (1100-LP)	5 mg/ ml	25	1100 LP	**Supplementary Table 2**
Figure 3c (1300-LP)	5 mg/ ml	50	1300 LP	
Figure 4a (1100-LP)	5 mg/ ml	50	1100LP	1000
Figure 4a (1300-LP)	5 mg/ ml	50	1300LP	1000
Figure 4a (1400-LP)	5 mg/ ml	50	1400LP	1000
Figure 5b	N/A	N/A	N/A	1000
Figure 6a	5 mg/ ml	50		1000
Figure 7b	5 mg/ ml	50		1000

Supplementary Table S2. the integration time (ms) used for each fluorescence image of phantom study

Depth (mm)	IR-TPE Pdots	
	1100-LP	1300-LP
1	1000	1000
2	2000	2000
3	3000	3000
4	4000	4000
5	5000	5000
6	6000	6000
